# Place of birth, duration of residence, neighborhood immigrant composition and body mass index in New York City

**DOI:** 10.1186/1479-5868-5-19

**Published:** 2008-04-06

**Authors:** Yoosun Park, Kathryn M Neckerman, James Quinn, Christopher Weiss, Andrew Rundle

**Affiliations:** 1 School for Social Work, Smith College, Northampton MA, USA; 2 Institute for Social and Economic Research and Policy, Columbia University, New York NY, USA; 3 Department of Epidemiology, Mailman School of Public Health, Columbia University, New York NY, USA

## Abstract

**Background:**

Past research has suggested that changes in culture explain the substantial weight gain seen in many immigrant groups with length of residence in the U.S. and across generations of residence in the U.S. However, it has been theorized that those settling in immigrant and co-ethnic neighborhoods may be buffered against this acculturative process and will be more likely to maintain home country dietary and physical activity patterns. To investigate this theory we incorporated measures of neighborhood immigrant composition into analyses of individual's body mass index (BMI) and generation of immigration and duration of residence in the U.S.

**Methods:**

Multilevel analyses were performed using objectively measured height and weight and survey data on diet and physical activity from a sample of 13,011 residents of New York City. Census data were used to calculate the proportion of foreign-born residents and extent of household linguistic isolation in a ½ mile radial buffer around the subject's home.

**Results:**

Foreign birth was associated with a significantly lower BMI (-1.09 BMI units, P < 0.001). This association was weakest among Asians (-0.66 BMI units, P = 0.08) and strongest among Black-Caribbeans (-1.41 BMI units, P = 0.07). After controlling for individual level variables, neighborhood proportion foreign-born was not associated with BMI, but increasing neighborhood linguistic isolation was inversely associated with BMI among Hispanics (-2.97 BMI units, P = 0.03). Furthermore among Hispanics, the association between foreign birth and BMI was stronger in low linguistic isolation neighborhoods (-1.36 BMI units, P < 0.0001) as compared to in high linguistic isolation levels (-0.42 BMI units, P = 0.79). Increasing duration of residence in the U.S. was significantly associated with higher BMI overall and among Hispanics.

**Conclusion:**

The analyses suggest that acculturation is associated with weight gain, and that neighborhood characteristics are only associated with BMI among Hispanics. However, we suggest that changes in body size currently interpreted as post-migration effects of acculturation to U.S. norms may in fact reflect changes in norms that are taking place internationally.

## Background

Although rates of overweight and obesity have risen for all population groups in the U.S., racial and ethnic minority populations, specifically Black Americans, Hispanics, and Native Americans, face a particularly high risk of obesity and attendant health problems even when socioeconomic status is controlled [1-4]. At the most proximal level, an individual's weight is influenced by caloric intake and physical activity [5,6]. However, more distal factors including cultural attitudes and practices have also been found to be linked to obesity trends. Changes in culture are believed to explain the substantial weight gain seen in many immigrant groups with length of residence in the U.S. and across generations of residence in the U.S.[2,7-9]. In immigrant groups, the factor of culture, or more precisely, acculturation – the process through which immigrants adopt the norms, behaviors and practices of the dominant culture post-immigration – has been identified as a particularly salient arena for study regarding body size [2,7]. Acculturation to U.S. patterns of increased sedentary behavior and poor dietary patterns may be reflected in obesity trends among immigrants [10,11]. Furthermore, differential rates of weight-increase among immigrant group may be due to differential rates of acculturation [2].

A commonly used proxy for acculturation is an immigrant's duration of residence in the host country [12,13]. Where health outcomes are concerned, the longer an immigrant lives in the U.S., the more he or she is assumed to adopt the normative (and potentially unhealthy) behaviors of the U.S. Increasing acculturation, as proxied by duration of residence in the U.S., is also assumed to indicate a loss of the "protective health behaviors associated with many immigrants' native cultures [9]." Loss of such factors as "attachments to supportive networks in the society of origin" involving loss of healthy behaviors and its replacement with less healthy behaviors, is thought to be detrimental to the health of immigrants [9]. While acculturation is regarded as a key influence on BMI and obesity, past studies of obesity among immigrants have neglected the neighborhood context, generally focusing on individual level measures of acculturation and not on the extent to which the individual lives in a more or less acculturated community or neighborhood.

Several studies have shown that first-generation immigrants have a lower body size than second- and third-generation immigrants, and that among immigrants, length of residence in the U.S. is associated with increased body size [2,7-9]. For instance, Singh and Siahpush found that immigrants' risks of negative health outcomes, including obesity, were lower than those for U.S. born populations but increased with length of residence in the country [14]. Kaplan and colleagues also found that recent Hispanic immigrants are generally healthier than the U.S.-born population, and that this distinction tends to diminish over time as immigrants adapt to a different sociocultural environment [9]. Goel and colleagues found that length of residence in the U.S. is associated with higher BMI among immigrants resident in the U.S. for 10 years or more, and that obesity rates among immigrants who have been in the country for at least 15 years approach those of native-born U.S. residents [11]. While Lauderdale and Rathouz found a low prevalence of overweight among Asian Americans, they also found the prevalence of overweight to be higher for those with longer length of residence in the U.S. [15].

In addition to nativity and duration of residence, language usage is one of the most commonly used proxy measures of acculturation [12,13]. Sundquist and Winkleby have shown, for instance, that English language usage is associated with abdominal obesity in a national sample of Mexican-American men and women, [7]. In a study of acculturation among immigrant Blacks, a high level of language acculturation was associated with a significantly lower odds of obesity [16]. But English fluency has not always had a positive association with obesity. In a study conducted within the Hispanic Health and Nutrition Examination Survey, Khan and colleagues found preference for English language to be associated with a lower BMI among Mexican women [17].

As noted by Cabassa, acculturation-related contextual factors above and beyond individual level measures of acculturation may influence how individuals adapt to a new culture [18]. Immigrant identity, for instance, has been found to be bolstered by residence among co-ethnics [19], and residence in a predominantly immigrant neighborhood is positively associated with a number of positive health outcomes among Latinos [20-23]. With few exceptions [2,24], however, the acculturation literature has tended to focus on the nativity, length of residence and language usage of the individual and/or the individual's family and neglected the larger community level immigration or acculturation context [13,18]. Past research has found that Hispanic immigrants are more likely than US-born Hispanics to live in neighborhoods with other immigrants and in greater neighborhood linguistic isolation [2], and residence in a primarily immigrant and co-ethnic neighborhood is likely to be associated with access to grocery stores and restaurants selling familiar home-country foods [25]. These findings suggest that neighborhood contexts are important to consider in relationship to obesity.

An additional concern with the literature is that many studies focus on single ethnic groups, making it difficult to know whether acculturation and BMI are related differently across ethnic groups. Here we address both lacunae in the literature with an analysis of body mass index (BMI) among native- and foreign-born individuals living in New York City. In addition to considering associations between BMI and generation and years of residence, we also examine how these associations change when neighborhood context variables, such as immigrant density (proportion foreign-born) and neighborhood linguistic isolation, are added to the model. For Hispanics, we also test for interactions between individual's place of birth and neighborhood immigrant density and linguistic isolation.

New York City is a unique context in which to study the associations between immigration, acculturation, and body size. It is a city of immigrants: of its 8 million residents, 36% are foreign-born. Of this foreign-born population, 43% entered the U.S. within the last 10 years, and 46% speak a language other than English at home [26]. Its large ethnic enclaves such as Chinatown (largely Chinese), Washington Heights (largely Dominican), Brighton Beach (largely Russian), and Flushing (largely Korean) provide the opportunity to make comparisons across a diverse set of immigrant populations without large-scale confounding by rural/urban or regional differences.

## Methods

Analyses of BMI and generation of immigration and duration of residence in the U.S. were conducted using data from volunteers who took part in a health survey conducted from January 2000 to December 2002 in all five of New York City's boroughs (Bronx, Brooklyn, Manhattan, Queens, and Staten Island) [27,28]. The survey was conducted by the New York City government, through the Academic Medicine Development Company (AMDeC), and has been described extensively elsewhere [27,28]. Data collection took place at six community-based health centers, two community hospitals, and six medical centers, and through the New York Blood Center. Research staff conducted extensive recruitment efforts in community settings such as health and neighborhood fairs, and the study was widely publicized in the city to encourage participation [27]. Volunteers enrolled in the study were measured for height and weight using clinical scales and rigid stadiometers available at the clinical locations where study subjects were enrolled. Our analysis includes 13,102 study subjects whose addresses could be geocoded to New York City. This sample is demographically and geographically representative of the population of New York City as ascertained by the Census and city-wide surveys conducted by the Department of Health [28]. The Columbia University IRB approved the analyses of demographic and neighborhood variables.

The survey collected information on place of birth and duration of residence in the U.S. Although Puerto Rico is part of the U.S., the questionnaire listed both the U.S. and Puerto Rico as options for place of birth. Given the very strong sense of ethnic identity among New York City Puerto Ricans, it is likely that island-born individuals indicated Puerto Rico rather than the U.S. as their place of birth [29-31]. Consistent with the Pew Hispanic Center National Survey of Latinos, individuals who indicated that they were born in Puerto Rico were classified as foreign-born [31]. The questionnaire asked all respondents to indicate how long they had lived in the U.S. with the following categories listed as choices: <5 years, 5–9 years, 10–14 years, 15–24 years and 25 or more years. Of respondents indicating they were born in the U.S., 99% chose the 25 or more years category.

Six racial and ethnic categories were distinguished: Asian American, Black – African American, Black – Caribbean American, Caucasian, Hispanic, and Other. Data on age, gender, weight, height, pre-tax income, and educational attainment were available for this analysis. Educational attainment was coded as eighth grade or less, some high school, high school graduate, vocational school, some college, college graduate, and graduate school.

Study subjects' home addresses were geocoded using ESRI's ArcGIS software and the immediate neighborhood in a half mile radial buffer around the home was described using U.S. Census 2000 summary file 3 data. The boundaries of the half mile radial buffers were spatially intersected with Census Block Group boundaries. Block groups in New York City are quite small, and an average of 33 block groups fell within a typical half mile radius circle. Census data from block groups fully or partially within the radial buffer were aggregated to calculate the following neighborhood descriptors: proportion of Asian residents, proportion of Black/African American residents, proportion of Hispanic residents, proportion of foreign-born residents, proportion of linguistically isolated households, and proportion of residents below the poverty line. The proportion of foreign-born residents was calculated such that individuals born in Puerto Rico were counted as foreign-born. For block groups that partially fell within the radial buffer the Census data were weighted in the calculations by the proportion of Block group area that fell within the buffer.

Multilevel analysis was employed because it allows for the simultaneous estimation of the effects of group-level and individual-level factors, and because it accounts for non-independence of observations that can occur within neighborhoods [32]. Statistical analyses of the cross-sectional baseline data were performed using SAS Proc Mixed [32]. To correct estimated standard errors for non-independence among subjects living within regions of New York City, Community Districts were used as a level two clustering variable. These Districts correspond to named New York City neighborhoods, such as China Town or East Harlem. No neighborhood measures at the Community District level were entered into the model as predictors; all neighborhood measures were modeled for ½ mile radial buffers around the subject's homes. Analyses were conducted first for the entire study population and then separately by racial/ethnic strata. Although all subjects were included in the pooled analysis, separate analyses were not performed for the race/ethnicity category of "other", because of insufficient cases, or for the Black – African American category, because the foreign-born subjects were few in number and very heterogeneous in their regions of origin. All models controlled for age, gender, education and neighborhood poverty rate, and the pooled analyses included indicators for five race/ethnic categories with Caucasian as the reference category. In analyses of Hispanics, country/territory of origin was also controlled for with subjects coded as being Puerto Rican, Dominican or other, with U.S. born Hispanics coded based on their father's country/territory of origin. Coding for other places of origin was not possible due to sparse cell counts in either the U.S. born or foreign-born categories. Analyses first considered individual-level characteristics alone, then added variables describing the immigrant composition of the study subject's immediate neighborhood.

Among Hispanics, we also examined interactions between individual-level and neighborhood-level characteristics by comparing residents of neighborhoods with a high or low proportion of foreign-born residents, and a high or low proportion of linguistically isolated households. Individuals were classified as living in high or low category neighborhoods by dividing them at the 75^th ^percentile of the distribution of proportion foreign-born, equivalent to living in a neighborhood with 44% foreign-born or more. Likewise, individuals were categorized into high and low linguistically isolated neighborhoods by dividing them at the 75^th ^percentile of proportion linguistically isolated, equivalent to living in a neighborhood with 25% or more linguistically isolated households. The association between place of birth and BMI was assessed separately among those living neighborhoods with high and low immigrant density and with high and low linguistic isolation. Additionally, interaction models were fit with cross-product terms for place of birth and the relevant neighborhood characteristic.

## Results

Information on place of birth was available from 13,011 of the 13,102 subjects with complete data for other important variables, and 33% of these individuals were foreign-born. Overall, for all Census tracts in New York City, the average proportion of residents who are foreign-born is 0.34, the average proportion of households who are linguistically isolated is 0.14 and the average poverty rate is 0.20. Compared to U.S. born subjects, foreign-born subjects lived in neighborhoods with a larger proportion of foreign-born residents, a greater proportion of people below the poverty line and a higher proportion of linguistically isolated households. Table 1 presents the demographic characteristics of the study population. After controlling for age, gender, race/ethnicity, and education, foreign birth was associated with a significantly lower BMI (see Table 2); income had no significant association with BMI once education was added to the model, and was therefore excluded. When the sample was stratified by race/ethnicity, foreign birth was significantly associated with a lower BMI in each stratum except among Asians where the difference narrowly escaped significance (p = 0.08). The difference in BMI by place of birth was smallest among Asians and largest among Black Caribbeans. When neighborhood immigrant composition and linguistic isolation were added to the models, the association between BMI and individual place of birth did not change. Immigrant composition was not associated with BMI in the overall study population, or in any of the racial/ethnic subgroups. Increasing linguistic isolation was significantly inversely associated with BMI among Hispanics, but not among Asians or in the over all study population (see Table 2). As expected, for the two groups likely to speak English in their home countries, Black – Caribbeans and Caucasians, linguistic isolation did not predict BMI. Analyses among Hispanics were repeated controlling for proportion of residents who were Hispanic and separately for Spanish language linguistic isolation, and neither of the variables were associated with BMI or altered the association between place of birth and BMI. Likewise among Asians, analyses were repeated controlling for proportion of residents who were Asian and separately for Asian language linguistic isolation; neither were associated with BMI or altered the association between BMI and place of birth. Among Black-Caribbeans, proportion of residents who were Black/African American in the neighborhood was positively but not statistically significantly associated with BMI (Beta = 1.63, p = 0.06).

**Table 1:** Demographic characteristics of the Study Population

Continuous variables	U.S.-born N = 8705	Foreign-born N = 4306
Age	Mean = 45.86, Median = 45.00, SD = 10.67	Mean = 47.00, Median = 47.00, SD = 10.32
Proportion of residents of the neighborhood below the poverty line the neighborhood.	Mean = 0.18, Median = 0.15, SD = 0.11	Mean = 0.22, Median = 0.20, SD = 0.11
Proportion of residents in the neighborhood who are foreign-born	Mean = 0.29, Median = 0.26, SD = 0.14	Mean = 0.41, Median = 0.41, SD = 0.16
Proportion of households in the neighborhood that are linguistically isolated	Mean = 0.12, Median = 0.9, SD = 0.09	Mean = 0.19, Median = 0.19, SD = 0.11
Categorical variables	N, (row %)	N, (row %)
Gender		
Female	5,452 (64.66)	2,980 (35.34)
Male	3,253 (71.04)	1,326 (28.96)
Race and Ethnicity		
Asian	96 (6.27)	1,434 (93.73)
Black – African American	1,737 (95.39)	84 (4.61)
Black – Caribbean	76 (11.91)	562 (88.09)
Caucasian	5,424 (88.28)	720 (11.72)
Hispanic	1,200 (45.87)	1,416 (54.13)
Other	172 (65.65)	90 (34.35)
Education		
Less than High School	96 (13.28)	627 (86.72)
Some High School	533 (57.37)	396 (42.63)
High School Graduate	1,989 (68.93)	894 (31.07)
Vocational School	121 (40.33)	179 (59.67)
Some College	2,108 (75.61)	680 (24.39)
College Graduate	2,075 (67.48)	1,000 (32.52)
Graduate School	1,789 (77.15)	530 (22.85)

**Table 2:** Associations^1 ^between BMI, Individual's Nativity and Neighborhood Level Immigrant Context

	All subjects		
	Model 1 Beta^2^, 95% CI, P-value	Model 2 Beta^2^, 95% CI, P-value	Model 3 Beta^2^, 95% CI, P-value

Individual foreign-born	-1.09 (-1.37, -0.81) p-value: <0.0001	-1.10 (-1.38, -0.82) p-value: <0.0001	-1.08 (-1.36, -0.80) p-value: <0.0001
Neighborhood proportion foreign-born	-	0.49 (-1.49, 0.52) p-value: 0.34	-
Neighborhood proportion linguistically isolated	-	-	-0.42 (-2.11, 1.28) p-value: 0.63

Asians Alone			

Individual foreign-born	-0.66 (-1.40, 0.07) p-value: 0.08	-0.69 (-1.42, 0.05) p-value: 0.07	-0.64 (-1.38, 0.09) p-value: 0.09
Neighborhood proportion foreign-born	-	0.41 (-0.87, 1.68) p-value: 0.53	-
Neighborhood proportion linguistically isolated	-	-	-1.09 (-3.11, 0.94) p-value: 0.29

Hispanics Alone			

Individual foreign-born	-1.17 (-1.67, -0.67) p-value: <0.0001	-1.12 (-1.63, -0.62) p-value: <0.0001	-1.10 (-1.61, -0.60) p-value: <0.0001
Neighborhood proportion foreign-born	-	-1.03 (-2.62, 0.56) p-value: 0.20	-
Neighborhood proportion linguistically isolated	-	-	-2.97 (-5.67, -0.28) p-value: 0.03

Black-Caribbeans Alone			

Individual foreign-born	-1.41 (-2.92, -0.11) p-value: 0.07	-1.55 (-3.01, -0.03) p-value: 0.05	-1.42 (-2.94, 0.10) p-value: 0.07
Neighborhood proportion foreign-born	-	3.14 (-0.65, 6.93) p-value: 0.10	-
Neighborhood proportion linguistically isolated	-	-	-0.80 (-8.48, 6.88) p-value: 0.84

Caucasians Alone			

Individual foreign-born	-0.95 (-1.40, -0.50) p-value: <0.0001	-0.93 (-1.38, -0.48) p-value: <0.0001	-0.95 (0.50, 1.40) p-value: <0.0001
Neighborhood proportion foreign-born	-	-1.19 (-2.39, 0.00) p-value: 0.05	-
Neighborhood proportion linguistically isolated	-	-	0.24 (-2.76, 3.24) p-value: 0.88

1 All models control for age, gender and education, and the models that includes all subjects also controls for race/ethnicity. The models for Hispanics also control for Hispanic ethnicity. Model 1 includes only individual level variables. Model 2 includes individual level variables, neighborhood proportion foreign-born and proportion of households in the neighborhood below the poverty line. Model 3 includes individual level variables, neighborhood proportion linguistically isolated and proportion of households in the neighborhood below the poverty line.

2 The Beta coefficients for foreign-born represents the adjusted difference in BMI for foreign-born versus U.S.-born individuals. The Beta coefficients for proportion foreign-born and proportion linguistically isolated represent the estimated difference in BMI for a 1 unit difference in these variables.

Table 3 shows the association between years of residence in the U.S. and BMI using US-born individuals as the referent group. Overall, and among Hispanics and Caucasians, there was a significant positive association between increasing years of residence in the U.S. and BMI. Among Asians, there was a very modest association between duration and BMI that approached statistical significance (p = 0.07). Among Black – Caribbeans, the overall trend was not statistically significant, although Black Caribbean immigrants residing in the U.S. for less than five years had significantly lower BMI. Controlling for neighborhood immigrant density and linguistic isolation did not alter the results for years of residence and the beta coefficients for linguistic isolation and proportion foreign-born were similar to those seen in the analyses of place of birth (results are not shown, but are available upon request). As in the analyses of place of birth, proportion foreign-born was not associated with BMI and linguistic isolation was only associated with BMI among Hispanics (beta = -2.83, p = 0.05).

**Table 3:** Association^1 ^between duration of residence in the U.S. and BMI

	All Subjects Beta^2^, P-value	Asians Beta^2^, P-value	Black – Caribbean Beta^2^, P-value	Caucasians Beta^2^, P-value	Hispanics Beta^2^, P-value
US Born	Ref	Ref	Ref	Ref	Ref

25+ of residence	-0.78, <0.0001	-0.63, 0.14	-1.88, 0.03	-0.55, 0.05	-0.88, 0.002
15–24 years of residence	-1.15, <0.0001	-0.50, 0.21	-1.26, 0.14	-1.68, 0.01	-1.01, 0.02
10–14 years of residence	-1.42, <0.0001	-0.78, 0.06	-1.45, 0.14	-1.28, 0.04	-1.97, 0.0002
5–9 years of residence	-1.27, <0.0001	-0.84, 0.05	-0.61, 0.60	-1.07, 0.05	-2.44, 0.0003
<5 years of residence	-1.59, <0.0001	-0.79, 0.06	-4.53, 0.004	-1.49, 0.03	-2.21, 0.003
Trend	-0.34, <0.0001	-0.11, 0.07	-0.27, 0.20	-0.35, <0.0001	-0.54, <0.0001

1 All models control for age, gender and education, and the models that includes all subjects also controls for race/ethnicity. The models for Hispanics also control for Hispanic ethnicity.

2 The Beta coefficients represent the adjusted difference in BMI between the respective categories of duration of residence and U.S.-born individuals.

P for difference in trend between Asians and Hispanics = <0.0001.

P for difference in trend between Asians and Caucasians = 0.22

For Hispanics, we assessed the possibility that the association between place of birth and BMI varied by neighborhood immigrant composition or linguistic isolation. Hispanics were categorized as to living in neighborhoods with high or low foreign-born populations and into neighborhoods with high and low linguistic isolation and the associations between individual place of birth and BMI was assessed by strata. Regardless of neighborhood immigrant composition, foreign-born Hispanic had a lower BMI than U.S.-born Hispanics (see Table 4). Overall, individuals with the highest BMI were those born in the U.S., who lived in neighborhoods with fewer immigrants and lower levels of linguistic isolation. The association between place of birth and BMI was similar in neighborhoods with high and low proportions of foreign-born residents. However, the association between place of birth and BMI was stronger in neighborhoods with lower levels of linguistic isolation, and was weaker in neighborhoods with high levels of linguistic isolation, although the interaction term for place of birth and neighborhood linguistic isolation status was not statistically significant (P = 0.27). We also examined whether the association between years of residence and BMI varied by neighborhood immigrant composition or by level of linguistic isolation, but found no evidence of interaction effects (results not shown).

**Table 4:** Adjusted^1 ^Mean BMI by Place of Birth and Neighborhood Characteristics for Hispanics

	US Born Adjusted mean BMI, (95% CI) N	Foreign-born Adjusted mean BMI, (95% CI) N	P-value for row		US Born Adjusted mean BMI, (95% CI) N	Foreign-born Adjusted mean BMI, (95% CI) N	p-value for row
High^2 ^proportion of foreign-born residents in neighborhood	28.99 (29.81–28.53) 228	27.95 (28.53–27.38) 427	0.03	High^3 ^proportion of linguistically isolated households in neighborhood	28.64 (29.56–27.72) 226	28.22 (28.86–27.59) 430	0.79
Low proportion of foreign-born residents in neighborhood	29.51 (29.98–29.04) 972	28.32 (28.71–27.93) 989	<0.0001	Low proportion of linguistically isolated households in neighborhood	29.64 (30.11–29.18) 974	28.28 (28.67–27.89) 986	<0.0001
P-value for interaction between place of birth and neighborhood characteristics		0.85				0.27	

1 adjusted for age, gender, education, Hispanic ethnicity and neighborhood poverty rate.

2 High is defined as being above the 75% percentile of the distribution of proportion foreign-born in all neighborhoods in the sample. The 75% percentile is equivalent to 0.44 of residents in the neighborhood being foreign-born.

3 High is defined as being above the 75% percentile of the distribution of proportion of households in the neighborhood being linguistically isolated in all the neighborhoods in the sample. The 75% percentile is equivalent to 0.25 of the households being linguistically isolated.

## Discussion

Consistent with findings from prior studies, place of birth and duration of residence in the U.S. predict BMI among residents of New York City overall, but with some differences in associations by race/ethnicity [9,11,14,15,33]. Associations between place of birth and BMI are strongest for Blacks from the Caribbean, intermediate for Caucasians and Hispanics, and weakest for Asians. The borderline statistical significance for Asians and Blacks from the Caribbean probably reflects the small numbers of U.S.-born subjects included in analyses for these two groups. In the overall study population, immigrants who had lived in the U.S. for the shortest duration had the lowest mean BMI score, and there was a significant trend across duration of residence categories. Across racial/ethnic groups, the trend of higher mean BMI score with increased duration of residence was strongest and most consistent among Hispanics. The trend was less consistent among Black – Caribbeans and Caucasians.

Similar to the finding for place of birth, the association between duration of residence in the U.S. and BMI was weakest among Asians. But the modest associations of borderline statistical significance among Asian may, nevertheless, be important. Research suggests that Asians have a higher percentage of body fat for a given BMI compared to Caucasians, and that the threshold for developing obesity and nutrition related non-communicable diseases (NR-NCD) in Asians occurs at a lower BMI level than for other populations [34]. Thus, even the modest increase in BMI among Asians associated with place of birth and length of residence in the U.S. seen in our sample may be of public health significance.

Findings such as ours have been generally interpreted to suggest that the U.S. has an obesogenic environment, that is, U.S. norms favor a positive energy balance and weight gain as compared to the home countries of the immigrants [2,9,11,15]. Being U.S.-born is associated with a significantly higher BMI, and greater length of residence in the U.S. is associated with higher BMI, because, it is thought, both variables capture aspects of an individual's level of acculturation to that obesogenic environment [9,11]. In order to better understand the factors that comprise this acculturative process, we included analyses of neighborhood measures of immigrant composition. We examined whether the proportion of foreign-born residents and proportion of linguistically isolated residents living in an individual's home neighborhood was associated with BMI over and beyond the individual's place of birth and length of residence in the U.S. Prior literature on linguistic isolation has predominantly investigated the language usage of the study subject and their family, not the extent to which residents of the neighborhood the subject lives in utilize English [2].

If, as the literature indicates, increased acculturation to an obesogenic environment has a detrimental health effect on immigrants, then neighborhood/community factors that retard that process of acculturation should be health promoting. It has been suggested, for instance, that disease patterns among immigrants who retain food consumption patterns of their country of origin remain more consistent with those of the home country than that of the U.S. [35,36]. If, as Gordon-Larsen and colleagues theorize, that "Where immigrants settle has substantial implications for dietary and activity patterns, given availability of markets that supply foreign versus American goods and services [2]," then residence in neighborhoods with high concentrations of immigrants should reduce the influence of U.S. dietary and physical activity norms that are thought to comprise the obesogenic environment of the U.S. [2]. Although their study of adolescent body size is not completely congruent with ours, Gordon-Larsen and colleagues analyzed proportion foreign-born and proportion Hispanic as measures of neighborhood level acculturation status but did not find these variables to be associated with overweight [2]. They also evaluated neighborhood linguistic isolation but did not include the variable in the final regression models [2]. Despite these measures not being significantly predictive of overweight status themselves, the inclusion of the acculturation variables in the model did reduce the effect of generation of immigration in Puerto Ricans and Cubans [2]. Another study utilized Census tract level proportion Hispanic as a predictor of adult BMI among Mexican-Americans, but after control for measures of neighborhood level socio-economic status this measure of neighborhood immigrant composition was not associated with BMI [24]. Among Caucasians, increasing neighborhood proportion foreign-born was associated with lower BMI with borderline statistical significance, suggesting that living in an immigrant neighborhood is protective for this group. Of the neighborhood context variables the only statistically significant finding was for neighborhood linguistic isolation being inversely associated with BMI among Hispanics. Predictably, for Caucasians and Black – Caribbeans, the two groups for whom linguistic isolation would not be thought to provide a protective effect, linguistic isolation was not associated with BMI. Additionally for Hispanics, in neighborhoods with high levels of linguistic isolation, place of birth was not associated with BMI, where as in neighborhoods with lower levels of linguistic isolation, there was a strong association between place of birth and BMI. Among Hispanics, those who were born in the U.S. and living in neighborhoods with low levels of linguistic isolation had the highest BMI scores. Similar to the two prior neighborhood studies investigating proportion Hispanic as a predictor, no association with proportion Hispanic was observed in the data. We believe that this is the first report to investigate whether there is statistical interaction between an individual's nativity and measures of neighborhood immigrant composition.

These findings are consistent with recent literature regarding acculturation and the "Hispanic Paradox", the observation of better health among first generation Hispanic immigrants [37,38]. The relative better health of the recent Hispanic immigrants is thought to be due to the group level maintenance of beneficial health behaviors practiced in the home country. As individuals acculturate to U.S. behavioral norms, the maintenance of home country health practices and their health status decline [37,38]. Communities with a high degree of linguistic isolation are more likely to be resistant to the adoption of U.S. norms and to maintain home country norms regarding body size. Consistent with this idea, our findings indicate that neighborhood level linguistic isolation is protective against increased body size, and particularly for U.S. born Hispanics, residence in less acculturated areas is beneficial. An interesting question our study raises, however, is why a similar neighborhood effect is not apparent among other immigrant groups. Among Hispanics, neighborhood effects occur because theoretically linguistic isolation signifies the presence of neighborhood characteristics that protect against health compromising behaviors. Why is this not true for other groups? For our sample, at least, the paradox of the neighborhood effect is why it is a Hispanic, rather than an immigrant, effect.

Overall, and among Hispanics, there were significant and strong positive associations between years of residence in the U.S. and BMI, but among Asians, the association between duration and BMI was very modest and of borderline statistical significance. This finding may be interpreted in several ways. Asians may, in fact, differ from other immigrant groups and not experience major weight gain despite lengthy residence in the U.S. However, past analyses of the 1992–1995 National Health Interview Study (NHIS) data on Asians showed that the odds of being overweight and obese increased with length of residence in the U.S. [15]. More recent analyses of the 2000 NHIS found results similar to those presented here: length of residence in the U.S. was associated with significant weight gain in Whites and Latinos, while among Asians weight gain with duration of residence was more modest and of borderline statistical significance [11]. Previous studies on BMI and immigration across ethnic groups have suggested that differences in weight gain across ethnic groups can be ascribed to differences in acculturation rates [2].

Conversely, the lack of association between duration of residence and BMI may represent cohort effects expressed in cross sectional data analyses. Like most prior work on length of residence and BMI, the analyses presented here are cross-sectional in nature and interpretation depends on the assumption that recent and earlier waves of immigrants had a similar BMI when they arrived in the U.S. However, if this assumption does not hold, home country trends could mask increases in BMI experienced by earlier waves of immigrants. For example, a recent nationally representative study in China showed a 50% increase in the prevalence of overweight and obesity from 1992 to 2002 [39]. Many Asians emigrate today from nations whose nutritional and physical activity profiles have changed dramatically in recent years, and it is possible that recent immigrants are arriving in the U.S. with higher BMI than did earlier cohorts. If this is indeed the case, even if the BMI of earlier cohorts has increased during their residence in the U.S., the gain would not be apparent in cross sectional analyses of duration of residence. Likewise, other cohort effects may obscure temporal trends in BMI when data are analyzed in a cross-sectional fashion as presented here. For instance, within the racial and ethnic classifications presented here, duration of residence may be associated with waves of immigration from different countries of origin that have different dietary and physical activity norms and in turn different BMI profiles. However, even with the large sample size available here, further analyses stratified by country of origin were not possible.

Particularly in an international city such as New York City, it maybe useful to think of the global epidemic of overweight and obesity as a cause of local trends in body size. Overweight and obesity has now become a global problem with a greater increase in obesity in lower- and middle-income developing countries than in high-income countries, and a world wide shift in the burden of obesity down the socio-economic scale [40-42]. The dominant models of acculturation which assumes acculturation as a post-migration process need to be reconsidered [43]. Recent immigrants come from home cultures which have already been profoundly affected by the influences of U.S. media and commerce, and have undergone significant economic and social changes which are reflected in shifts in diet and physical activity patterns. What is currently interpreted in the literature as post-migration effects of acculturation may in fact be a reflection of changes in norms that are taking place elsewhere.

Additionally, immigrants' post-migration contact with their countries of origin, and the shifts in their cultures of origin, must be considered. What researchers theorize as a linear process of acculturation to the U.S. might, in actuality, represent changes in immigrants' home culture transmitted to immigrants in the U.S. Foreign language media penetration in the New York City market is considerable. For example, during the 2005 July sweeps period, Univision, the Spanish language station with programs largely originating from South and Central America, drew more viewers than all other stations among 18–49 year olds, Hispanic or otherwise [44]. Whether garnered through media or through travels back to the country of origin, immigrants' post-migration acculturation may be affected by cultural shifts in that of the country of origin. That acculturation might be mediated – made more or less attractive or at least filtered and interpreted in various ways – through the home country's globalization rather than through direct experience, must be considered.

A number of methodological issues should be considered when interpreting these results. Socio-economic status was controlled for using information on educational status. Educational systems, however, vary across countries and thus educational attainment may not adequately control for confounding by socio-economic status, although income no longer predicts BMI once education is included in the regression model [28]. We also note that further control for neighborhood level proportion poverty did not alter the associations between BMI and place of birth or duration of residence. Additionally, individuals born in Puerto Rico, although U.S. citizens, were considered to be foreign-born in these analyses. This approach is consistent with analyses conducted by the Pew Hispanic Center, which notes that island born Puerto Ricans are born into a culture dominated by Spanish and their views and beliefs are much closer to foreign-born Hispanics than U.S. born Hispanics [31]. However as we note above, with globalization and the rise of Spanish language media reducing cultural distances, it is debatable as how best to classify island-born Puerto Ricans for analyses such as these. Unlike the Hispanic Health and Nutrition Examination Survey and other studies that sampled on, or over sampled for, country or territory of origin, we did not have sufficient numbers of individuals to finely categorize Hispanics into ethnic sub-groups. There is some evidence that the association between BMI and measures of acculturation varies across ethnic groups within the larger Hispanic designation [2,12]. While we were not able to perform stratified analyses as conducted elsewhere, our analyses did control for Puerto Rican, Dominican or other country of origin. Lastly as noted above, the study is limited by its cross-sectional design and inability to make prospective causal inferences.

In conclusion, a necessary component to understanding immigrant health is information on patterns of nutrition, physical activity, and body weight beliefs/values and behavior/practices in the immigrants' countries of origin. This information would allow for a comparative context against which immigrants' current practices and beliefs and perceptions of cultural beliefs and practices of their countries of origin can be understood. The compilation of relevant socio-demographic and epidemiological information from the immigrants' countries of origin would allow useful comparisons necessary to understanding immigrants' decision making processes and acculturative patterns in the U.S.

## Conclusion

As expected, foreign birth and duration of residence in the U.S. were associated with BMI and may suggest that immigrants alter their dietary and physical activity patterns in a manner that promotes weight gain. Contrary to theory there was little evidence that neighborhood immigrant context predicted BMI or strongly altered the apparent acculturative process. However, we suggest that the apparent differences in BMI between groups of different immigrant status or duration of residence may not represent a simple post-migration process, but may also need to be considered in terms of globalization and trans-national identity.

## Authors' contributions

YP conceptualized the data analyses and wrote the manuscript. KMN helped design the study and helped write the manuscript. JQ acquired the neighborhood level data, geocoded the subjects and created the neighborhood measures. CCW helped design the study and helped write the manuscript. AGR acquired the data human health data, conducted the statistical analyses and helped write the manuscript.
